# Supplementary material for: Effect of Combined Levothyroxine (L-T4) and 3-Iodothyronamine (T1AM) Supplementation on Memory and Adult Hippocampal Neurogenesis in a Mouse Model of Hypothyroidism
Source: Int J Mol Sci. 2023 Sep 8;24(18):13845. doi: 10.3390/ijms241813845 (PMC10530993; doi:10.3390/ijms241813845)
Supplement: Supplementary file 1 [file ijms-24-13845-s001.zip › ijms-2550442-supplementary.pdf]

Supplementary Materials

Effect of Combined Levothyroxine (L-T_4) and 3-Iodothyronamine (T_1AM) Supplementation on Memory and Adult Hippocampal Neurogenesis in a Mouse Model of Hypothyroidism

Grazia Rutigliano [1,2,3,*], Andrea Bertolini [2], Nicoletta Grittani [2], Sabina Frascarelli [2], Vittoria Carnicelli [2], Chiara Ippolito [4], Stefania Moscato [4], Letizia Mattii [4], Claudia Kusmic [3], Alessandro Saba [2], Nicola Origlia [5] and Riccardo Zucchi [2]

[1] Institute of Clinical Science, Imperial College London, London SW7 2AZ, UK
[2] Department of Pathology, University of Pisa, 56126 Pisa, Italy; a.bertolini2@student.unisi.it (A.B.); nicolettamaria.grittani@gmail.com (N.G.); sabina.frascarelli@unipi.it (S.F.); vittoria.carnicelli@unipi.it (V.C.); alessandro.saba@unipi.it (A.S.); riccardo.zucchi@unipi.it (R.Z.)
[3] CNR Institute of Clinical Physiology, 56124 Pisa, Italy; kusmic@ifc.cnr.it
[4] Department of Clinical and Experimental Medicine, University of Pisa, 56126 Pisa, Italy; chiara.ippolito@unipi.it (C.I.); stefania.moscato@unipi.it (S.M.); letizia.mattii@unipi.it (L.M.)
[5] CNR Institute of Neuroscience, 56124 Pisa, Italy; nicola.origlia@in.cnr.it
* Correspondence: grutigli@ic.ac.uk

Table of Contents

Supplementary Methods 2

HPLC-MS/MS analysis of thyroid hormones 2

Table S1. *Mass spectrometry operative parameters.* .... **Error! Bookmark not defined.**

Table S2: *Predesigned 96-well PrimePCR™ "Neurogenesis Tier 1 M96" collection panel (Bio-Rad, USA) containing primer sets for 88 gene targets involved in neurogenesis pathway* **Error! Bookmark not defined.**

Supplementary Results 5

Figure S1. *Elevated plus maze* .... **Error! Bookmark not defined.**

Figure S2. *Tail suspension test* .... **Error! Bookmark not defined.**

Supplementary Methods

HPLC-MS/MS analysis of thyroid hormones

Serum samples (100 µL) were placed in a 2 mL Eppendorf ® tube and added with 10 µL of a 200 ng/mL ($^{13}C_6$-T_3 and $^{13}C_6$-T_4) stable isotope-labelled internal standards mixture. Samples were gently vortexed and equilibrated for 30 min at room temperature (RT). Then, 300 µL of cold acetone were added and samples were vortexed and kept 30 min at 4°C to allow proteins precipitation. After a centrifugation step at 22780 x g for 10 min, the supernatants were transferred to a new 2 mL Eppendorf ® tube, warmed up at 40 °C, and dried under a gentle stream of nitrogen until reaching ~ 100 µL. Afterwards, samples were added with 400 µL of a 0.1 M potassium acetate buffer (pH=4) and submitted to a Solid Phase Extraction (SPE) using Agilent (Santa Clara, CA, USA) Bond-Elut Certify 130 mg SPE cartridges, as previously described [35]. Eluates were dried at 40 °C under a nitrogen stream, reconstituted with 100 µL of methanol/water (30/70 by volume) and 5 µL were injected into the HPLC-MS/MS system. Stock solutions of T_3 and T_4 were separately prepared at 1 µg/mL concentration in methanol. Calibration curves were daily prepared by serial dilution with methanol at a concentration ranging from 0.1 to 100 ng/mL. Water, methanol (MeOH), acetonitrile (ACN), and formic acid (FA, purity ≥ 98%-100%) were LC-MS grade, while ammonium hydroxide (NH4OH, 28% in H2O (w), purity ≥ 99.99%), 2-propanol, hexane, and dichloromethane were analytical grade. 3,3′,5-Triiodo-L-thyronine (T_3), T_3-$^{13}C_6$, L-Thyroxine (T_4), and T_4-$^{13}C_6$ solutions, all of them 100 µg/mL in MeOH with 0.1 M NH_4OH, and solvents were purchased from Sigma Aldrich-Merck (Germany).

The instrument layout consisted in an Agilent (Santa Clara, CA, USA) 1290 UHPLC system, including a binary pump, a column oven set at 20°C, and a thermostated autosampler, coupled to an AB-Sciex (Concord, Ontario, Canada) QTRAP 6500+ mass spectrometer working as a triple quadrupole, and equipped with an IonDrive™ Turbo V source. Chromatographic separation was achieved by using a 110 Å, 2x50 mm, 3µm particle size, Gemini C18 column (Phenomenex, Torrance, CA), protected by a C18 Security Guard Cartridge and using (A) MeOH/ACN (20/80 v/v) added with 0.1% FA and (B) water containing 0.1% FA as mobile phases. The integrated 6 ports switching valve was used to discard both head and tail of the HPLC runs. Gradient elution (400 µL/min flow rate) was performed as follows: 0.1-3 min (A) 5%, 8.5 min (A) 65%, 9.0-11.0 min (A) 100%, 11.50-13.50 (A) 5%. System control, data acquisition and analyses were performed using an ABSciex Analyst® version 1.7.3 software. A mass spectrometry selected reaction monitoring (SRM) method operated in positive ion mode. For each compound, after the optimization of declustering potential (DP), collision energy (CE) and collision exit potential (CxP), three transitions were considered in the analysis. Based on the highest signal/noise

ratios, one of them was used as quantifier (Q) and the other two as qualifiers (q) as reported in Table S1. Further operative parameters were gas source 1 (GS1), 60 arbitrary units; gas source 2 (GS2), 45 arbitrary units; ion spray voltage (ISV), 5.5 kV; source temperature (TEM), 650°C; entrance potential (EP), 10V; Curtain gas (CUR), 20 arbitrary units; collision gas (CAD) N_2, operative pressure with CAD gas on, 2 mPa.

Table S1. Mass spectrometry operative parameters.

Analyte	SRM transitions (Da)	Operative Parameters		
		DP (V)	CE (V)	CXP (V)
T_3	651.8 → 478.9 (q)	76	47.7	13.7
	651.8 → 508.0 (q)		31.2	14.8
	651.8 → 605.9 (Q)		31.3	17.9
$^{13}C_6$-T_3	657.8 → 484.9 (q)	76	47.7	13.7
	657.8 → 514.0 (q)		31.2	14.8
	657.8 → 611.9 (Q)		31.3	17.9
T_4	777.8 → 604.8 (q)	82	52.8	17.0
	777.8 → 633.9 (q)		36.0	18.6
	777.8 → 731.9 (Q)		34.0	22.0
$^{13}C_6$-T_4	783.8 → 610.8 (q)	82	52.8	17.0
	783.8 → 639.9 (q)		36.0	18.6
	783.8 → 737.9 (Q)		34.0	22.0

Table S2. Predesigned 96-well PrimePCR™ "Neurogenesis Tier 1 M96" collection panel (Bio-Rad, USA) containing primer sets for 88 gene targets involved in neurogenesis pathway.

	**1**	**2**	**3**	**4**	**5**	**6**	**7**	**8**	**9**	**10**	**11**	**12**
**A**	*Akt1*	*Bmp2*	*Ctnnb1*	*Epo*	*Gdnf*	*Igf1r*	*Mapk8*	*Nfkb1*	*Ntrk2*	*Rein*	*Sox2*	*Tbp*
**B**	*Akt2*	*Bmp4*	*Cxcl12*	*Esr1*	*Gfap*	*Jak2*	*Mapt*	*Ngf*	*Ntrk3*	*Rest*	*Src*	*Gapdh*
**C**	*Akt3*	*Casp3*	*Cxcr4*	*Esr2*	*Gsk3b*	*Kdr*	*Met*	*Ngfr*	*Pax6*	*Ret*	*Stat3*	*Hprt*
**D**	*Apoe*	*Cd44*	*Dcx*	*Fgf2*	*Hes1*	*Kit*	*Ncam1*	*Notch1*	*Pdgfra*	*Rhoa*	*Tgfb1*	gDNA
**E**	*App*	*Cdk5*	*Dll1*	*Fgfr1*	*Hif1a*	*L1cam*	*Nes*	*Nr3c1*	*Prom1*	*S100b*	*Th*	PCR
**F**	*Ascl1*	*Cnr1*	*Egf*	*Fgfr2*	*Hras1*	*Lep*	*Neurod1*	*Nrg1*	*Psen1*	*Shh*	*Trp53*	RQ1
**G**	*Bcl2*	*Cntf*	*Egfr*	*Foxg1*	*Ifng*	*Mapk1*	*Neurog1*	*Ntf3*	*Pten*	*Smad4*	*Vegfa*	RQ2
**H**	*Bdnf*	*Creb1*	*Ephb2*	*Gap43*	*Igf1*	*Mapk3*	*Neurog2*	*Ntrk1*	*Rb1*	*Sod1*	*Wnt1*	RT

gDNA, DNA Contamination Control

PCR, Positive Control

RQ1 and RQ2, Quality Probe

RT, Reverse Transcription Control

Supplementary Results

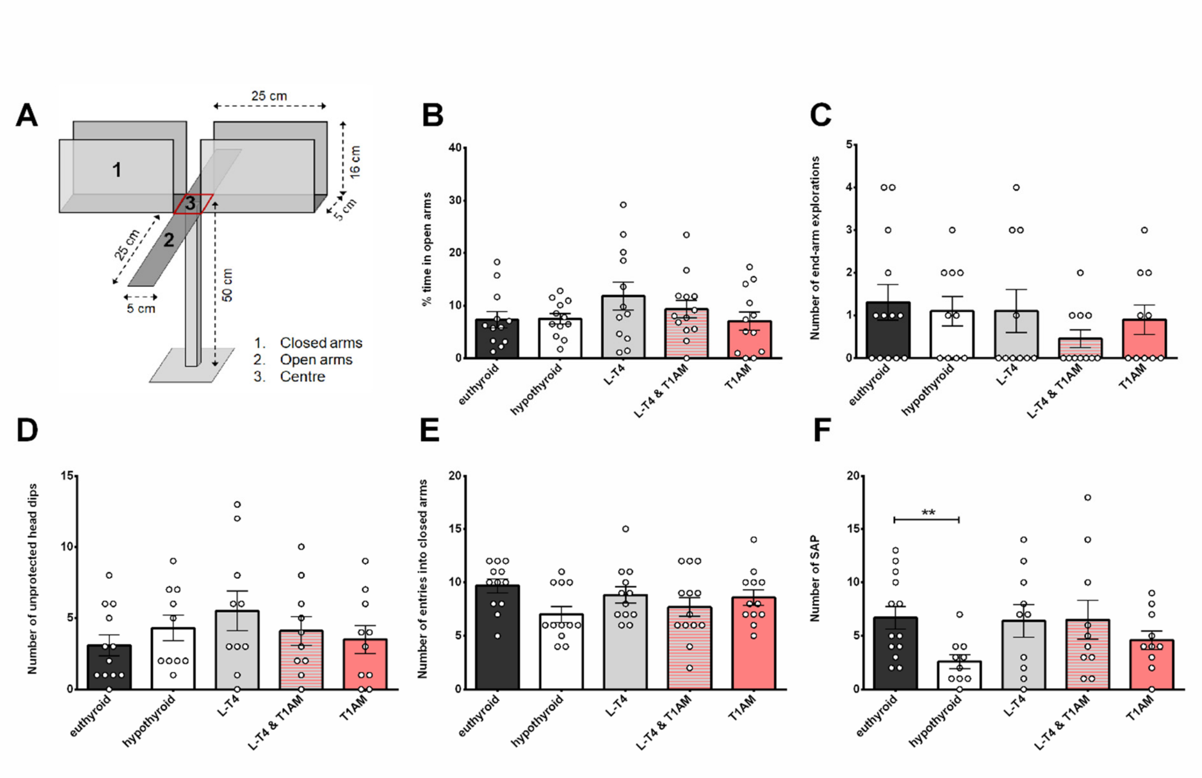

Figure S1. Elevated plus maze. **(A)** EPM apparatus. **(B-D)** Anxiety-related behaviours. **(E)** Spontaneous locomotor activity. **(F)** Risk-assessment and decision-making behaviours. Results are plotted as mean±SEM using Graph Pad Prism software. ANOVA and Tukey's multiple comparison test were used to evidence eventual significant differences between the different groups (** $p < 0.01$). Hypothyroid n=12, Euthyroid n=13, L-T_4 n=12, L-T_4+T_1AM n=13, T_1AM n=12.

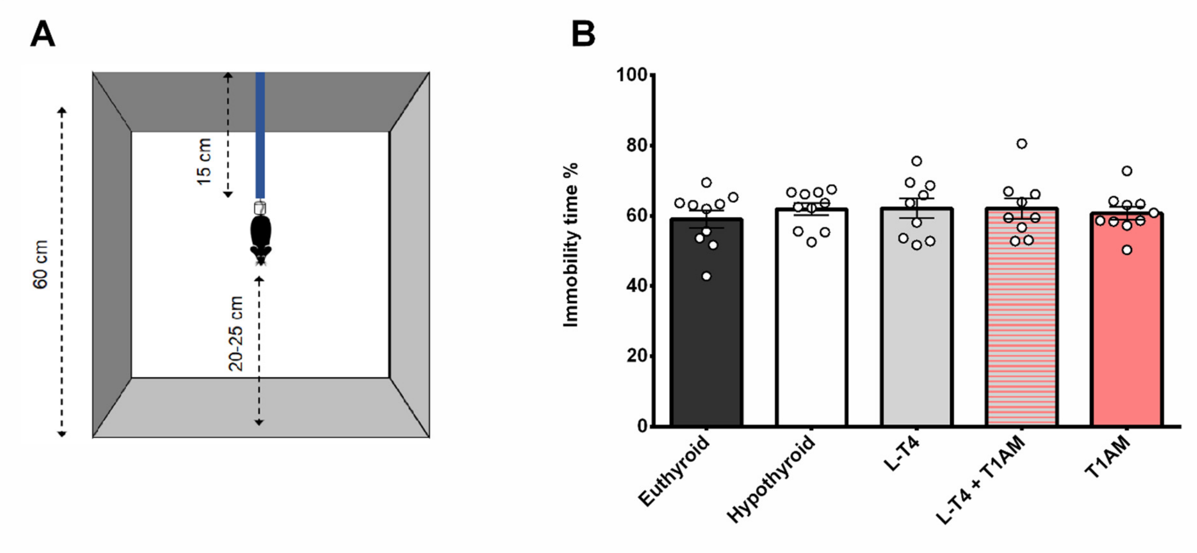

Figure S2. Tail suspension test. **(A**) TST apparatus. **(B)** Depression-related behaviour showing the % of immobility time among the different treatment groups. Results are plotted as mean±SEM using Graph Pad Prism software. Hypothyroid n=10, Euthyroid n=10, L-T_4 n=9, L-T_4+T_1AM n=9, T_1AM n=10.
